# Hemorrhoids screening and treatment prior to LVAD: is it a necessity?

**DOI:** 10.1186/s13019-016-0441-z

**Published:** 2016-04-12

**Authors:** Hadi Skouri, Mohammed Shurrab, Jad Zahnan, Samer Deeba, Pierre Sfeir, Walid Gharzuddin, Saleem Haj-Yahia

**Affiliations:** Division of Cardiology, Department of Internal Medicine, American University of Beirut Medical Center, Beirut, Lebanon; National Heart, Lung & Transplant Institute, An-Najah National University Hospital, An-Najah National University, Nablus, West Bank Palestine; Faculty of Medicine & Health Sciences, An-Najah National University, Nablus, West Bank Palestine; Department of Surgery, American University of Beirut Medical Center, Beirut, Lebanon; Institute of Cardiovascular and Medical Sciences, The University of Glasgow, Glasgow, Scotland, UK

**Keywords:** Hemorrhoids, LVAD, Screening

## Abstract

Continuous-flow left ventricle assist devices (CF-LVADs) has become an essential modality in the management of stage D heart failure (HF) with significant improvement in survival and quality of life. Due to the durability of such devices and long term support complications such as bleeding and aortic insufficiency has emerged. Bleeding accounts for more than 20 % with the majority being from the gastrointestinal tract. The increase of bleeding tendency are mainly attributed to the loss of large von Willebrand’s Factor (vWF) multimers due to shear stress with the chronic intake of anticoagulants. We are reporting two cases of patients with Stage D HF and history of hemorrhoids presenting for LVAD implantation. Many efforts that decrease bleeding related to CF-LVADs will be discussed with focus on hemorrhoids.

## Background

Continuous-flow left ventricle assist devices (CF-LVADs) has become an essential modality in the management of stage D heart failure (HF) with significant improvement in survival and quality of life. Compared with the older pulsatile devices, CF-LVADs has less complications, mainly infections and thrombosis [1–4], due to the durability of such devices and long term support. Other complications are emerging such as bleeding and aortic insufficiency. Bleeding is one of the major reported complications accounting for more than 20 % [4], with the majority being from the gastrointestinal tract [2], especially with the need of long-term anticoagulation therapy. The increase of bleeding tendency are mainly attributed to the loss of large von Willebrand’s Factor (vWF) multimers due to shear stress [4]. Many efforts are being implemented to decrease bleeding related to CF-LVADs [4].

We are reporting two cases of patients with Stage D HF and history of hemorrhoids presenting for LVAD implantation.

### Case 1

Fifty-five-year-old male with valvular cardiomyopathy referred to our institution with stage D HF and multi-organ failure. He was in INTERMACS profile I, in cardiogenic shock, on Intra-aortic balloon pump (IABP) and intra venous inotropes and pressors. He also suffered acute on top of chronic renal failure for which hemodialysis was initiated. His hospital mortality HeartMate II risk score reached 73 %.

Two weeks after decongestion with ultrafiltration, near optimization of nutritional status and normalization of pro-thrombin time, a HeartMate II LVAD was implanted as a bridge to decision.

One week post LVAD implantation, he developed severe lower gastrointestinal (GI) bleeding attributed to stage III hemorrhoids, which resulted in a hypovolemic shock necessitating multiple transfusions and withholding anticoagulation. In addition to lower GI bleeding, he was still supported by mechanical ventilation and hemodialysis. Due to profuse bleeding and necessity for long term anticoagulation, he underwent urgent hemorrhoidal artery ligation (HAL). Post surgery he developed hypotension with elevated pump flow, low pulse index and elevated pump power, suggestive of pump thrombosis. This led to further vascular collapse and right ventricular failure resulting in cardiopulmonary arrest with unsuccessful resuscitation.

### Case 2

Fifty-five-year-old male with dilated cardiomyopathy on optimal medical therapy and post CRT –D implantation, admitted with acute decompensated heart failure. He was in cardiogenic shock and stabilized thereafter with intravenous inotropes. He was inotrope-dependent (HF Stage D INTERMACS profile 3) and a candidate for a life saving CF-LVAD implantation. During preparation for the procedure he developed episodes of hematochezia. Workup showed bleeding from grade 3 internal hemorrhoids. HAL was performed prior to LVAD surgery. LVAD implantation was performed uneventfully. Hematochezia did not recur despite therapeutic anticoagulation. During a 2 year-follow up, the patient reported no GI bleeding.

## Discussion

As more than 5 million people have congestive heart failure and with the number projected to increase [5], LVAD became the standard of care for patients with heart failure refractory to optimal medical treatment and requiring circulatory support as a bridge to transplant, destination therapy or bridge to myocardial recovery [6].

In order to avoid device related thromboembolic events, it is recommended that patients with LVAD be maintained on antiplatelet, and therapeutic anticoagulation [7]. In addition LVAD patients are at increased risk of bleeding due to angiodysplasia and acquired vonWilleberand’s disease especially in continuous flow LVAD (CF-LVAD) [8]. In multiple reports angiodysplasia was found as the culprit lesion for GI bleeding. In continuous-flow LVADs, blood is directed out of the left ventricle directly into the aorta without passing through the aortic valve which results in less frequent opening of the valve causing a reduced aortic pulse pressure. This causes a reduction in the arteriole smooth muscle causing a dilation in mucosal veins and angiodysplasia formation [9]. Also Efficient platelet aggregation and adhesion depends on the high molecular weight (HMW) multimers of vWF. Similarly to aortic stenosis, high shear stress due to the passage of blood through continuous flow LVAD and biventricular pulsatile VAD results in the cleavage of HMW multimers, resulting in dysfunction vWF and acquired von Willerbrand’s disease [8–11]. Therefore bleeding events for these patients increase.

The incidence of upper and lower GI bleeding in both pulsatile and non pulsatile LVAD patients increased, **despite the advances in the hemodynamics and biocompatibility of these devices,** more notable in the non pulsatile group [5, 12].In a report by Harvey et al. the incidence of GI bleeding after LVAD implantation varied between 18–40 % [6]. Although in many reports it is unknown, the bleeding site can involve the entire gastrointestinal tract [13], with upper being more common than lower GI bleeding, mainly in the stomach, followed by duodenum, jejunum and esophagus [9]. Internal hemorrhoids, as a source of lower GI bleed is common, as reported in a case series by Siddiqui et al., to be the source of bleeding in 8 out of 38 cases [5].

With the increased incidence of GI bleeding following LVAD surgery, preventing this complication prior to embarking on implant might be reasonable, especially in patients at higher risk of bleeding.

In Case 1, patient had HAL post LVAD implant, yet this was complicated by cardiac arrest and death despite many efforts to resuscitate the patient. That being said, in Case 2 we opted for hemorrhoidal artery ligation (HAL) before LVAD implantation, despite the patient’s clinical status. Thus avoided any fatal complications related to bleeding or a second procedure (e.g. HAL) post LVAD implant.

HAL is an operation started in 1996 by Morinaga et al. for the surgical treatment of moderate grade and prolapsed hemorrhoids. It centers around the treatment of hemorrhoids by cutting off the blood supply with guidance from a Doppler probe [14]. Hence, this procedure is a low risk, less invasive and efficacious procedure, making it a good alternative to conventional haemorrhoidectomy or stapled haemorrhoidopexy in the short and medium term as endorsed by the National Institute for Health and Care (NICE) [15]. According to the 2014 ESC/ESA Guidelines on non-cardiac surgery, heart failure is a well-recognized factor for peri-operative and post-operative cardiac events, while patients with left ventricular ejection fraction (LVEF) <30 % might be at a greater risk [16]. However since HAL is less invasive than conventional open hemorrhoidectomy, it was the procedure of choice in our patient considering the potential for a related GI complication before LVAD implantation. It should be noted, that Jeong et al, in there one year follow up of patients post dopppler-guided HAL, noted a recurrence rate including re-bleeding of 14 %. However this large number was higher than previously published reports [17].

Secondary prevention strategies for GI bleed post LVAD implantation had been discussed, including treating Helicobacter pylori, daily proton pump inhibitor use, limiting non steroidal anti inflammatory use and sub cutaneous octreotide injection to treat angiodysplasia [9]. Also lowering pump speed was suggested as an approach to limit bleeding [18]. Based on the 2013 International Society for Heart and Lung Transplantation Guidelines for Mechanical Circulatory Support: “reducing the pump speed for continuous flow pumps in the setting of recurrent gastrointestinal bleeding due to arteriovenous malformations may be considered”. Considering that angiodysplasia is caused by reduced pulsatility, thus decreasing pump speed can result in increase pulsatility and decreased angiodysplasia formation [18]. Also repeated transfusion of vWF has been reported to control bleeding caused by angiodysplasia [10], however it is not recommended yet.

In our view many efforts should be made preoperatively to decrease bleeding complications related to LVAD. Some of these primary prevention measures include preoperative assessment of the coagulation profile, vitamin K administration in case of elevated INR levels with postponement of surgery 5 to 7 days to allow cessation of vitamin K antagonists and thineopyridines, screening for Heparin Induced Thrombocytopenia (HIT) and in some cases the use of recombinant factor VII [4]. Screening for common cause of bleed, such as hemorrhoids, prior to surgery as primary prevention had not been addressed previously. Hemorrhoids are recognized as a common cause of lower GI bleeding; however its true epidemiology is unknown. Its estimated prevalence is 4.4 % in the United States [19]. Being that common as a cause of GI bleeding, screening for hemorrhoids prior to LVAD surgery could prevent this complication especially that approximately 40 % of patients with hemorrhoids might be asymptomatic.

Since with the current available evidence, routine screening for hemorrhoids in asymptomatic patients might not be cost effective, our two cases raise the need for a multicenter study to shed light on this pathological entity and its management in the LVAD population, as a potential measure to reduce post LVAD bleeding complications.

## Conclusion

GI bleeding is a common complication in LVAD patient while hemorrhoids are a potential cause of mortality and morbidity.Since LVAD patients are in continuous need for therapeutic anticoagulation, screening and managing hemorrhoids in the advanced stages of HF maybe recommended to reduce the risk of potential bleeding and bleeding complications in LVAD patients. Further studies are required to assess the benefits and cost effectiveness of the suggested therapeutic intervention.
